# Trends and associations of remote workdays and short sickness absences among Finnish knowledge workers from 2019 to 2023

**DOI:** 10.1177/14034948251380639

**Published:** 2025-10-07

**Authors:** Annina Ropponen, Annu Haapakangas

**Affiliations:** 1 Finnish Institute of Occupational Health, Helsinki, Finland; 2 Karolinska Institutet, CNS, Division of Insurance Medicine, Stockholm, Sweden

**Keywords:** Telework, sick leave, cohort study, longitudinal, working time

## Abstract

**Aims:**

The aim was to investigate short (1–3 days) sickness absence (SA) and remote work in 2019–2023 among a cohort of Finnish knowledge workers. A specific aim was to investigate the role of working hours and the associations between remote work and SA and if the associations would differ before, during, or after the COVID-19 pandemic.

**Methods:**

Employer-owned register data of 5535 knowledge workers for working hours (daily and weekly working hours), remote workdays/week, and short, 1–3 days, SA from 2019 to 2023 were investigated with a fixed-effects Poisson regression analysis for incidence rate ratios (IRRs) with 95% confidence intervals (95%CI).

**Results:**

The overall associations between remote work and short SA indicated that each 1-day increase in remote workdays was associated with higher odds of short SA (IRR 1.27, 95%CI 1.24, 1.30). The comparison across the years 2019–2023 showed varying associations. In the pre-pandemic year, 2019, there was no statistically significant association between remote workdays and short SA. Since 2021, doing no remote work has been associated with a lower likelihood of short SA. Instead, working remotely for 1–2 days or 3–5 days/week was associated with higher likelihood only when compared with no remote work.

**Conclusions:**

**Among knowledge workers, remote work seems related to short, 1–3 days of SA only after the COVID-19 pandemic. The possibility of working remotely might be an important factor in mitigating infections, while our results raise the assumption that presenteeism might be prevalent in remote work.**

## Background

Remote work has already existed for decades [1] being defined as work that is fully or partially performed at an alternative location other than the typical place of work (e.g. the employer’s premises), based on the employer’s approval [2]. Due to the developments in digital infrastructures and devices, knowledge workers can increasingly perform their work using mobile technology not only in offices, but also while traveling, at customers’ premises, in public places, or remotely [3,4], and their working life has also changed dramatically over recent years due to the COVID-19 pandemic and the consequent increase in remote work [5,6]. On the other hand, the rates of sickness absence (SA), along with assumptions of sickness presenteeism among knowledge workers who can more or less work where and when they wish, have been under debate [7-10]. Until now, studies of SA and remote work among knowledge workers have not indicated associations between remote work with SA or toward lesser SA among those with the possibility to work remotely, but these studies have been hampered, as they have focused on the preceding or early years of the pandemic [11-13], or due to cross-sectional design and/or survey data [3,8,12,14]. Thus, studies using a longitudinal design and register data of remote work and SA are needed to explore this phenomenon [13,15]. Thus, as remote work may retain its role for knowledge workers, understanding the patterns of remote work and SA would help employers support their employees’ work capacity, productivity, and well-being.

SA is a known proxy for health status and reflects workability due to the linkage between short (1–3 or 1–5 days by self-certification) SA, that have been shown to predict longer SA [16,17]. Such a short SA is a common and fully compensated practice in Nordic countries. Short SAs are mainly utilized by the employees for health-related reasons, for example, epidemics or for symptoms such as coughing, headache, or migraine that hamper the work ability of the employees or might be contagious, such as stomach flu. On the other hand, short SA can reflect other causes than longer SA that require medical certification. For example, some evidence exists that short SA reflects self-perceived health with or without underlying disease [18]. Thus, we assumed that remote work can be a double-edged sword: Remote work may enable employees to work while having (mild) symptoms, thus avoiding contamination of colleagues in case of infectious diseases, while workload in remote work, especially when linked with long working hours or persisting symptoms or disease, may increase SA. Yet, the COVID-19 pandemic was very contagious. Thus, the regulations and instructions for remote work, symptoms of infectious diseases, and the availability of public transportation or employer premises have affected workers’ intentions, possibilities, or even permissions to work remotely [9,19], calling for assessments from the COVID-19 preceding years to their aftermath.

Yet another aspect might add complexity to the associations between remote work and SA. Working remotely might add flexibility to working hours. Such flexible working hours are known to be linked to better health, well-being, and work/life balance [20-22], while they are also known to have negative effects [23,24]. Furthermore, suggestions exist that flexible working hours should have limits that reflect, for example, the importance of the boundaries between work and leisure time [25]. Remote work has been suspected to lead to feelings of increased time pressure and stress, potentially due to a lack of support and contact, or unclear expectations of results [24]. Remote work may also lead to blurred boundaries between work and leisure time, and consequently to prolonged working hours [8], which have been linked with an increased SA risk [26-28]. An emergent need exists for studies elaborating on the associations between remote work and SA while accounting for the working hours, as such studies have been rare to the best of our knowledge.

This prospective cohort study aimed to investigate short (1–3 days) SA and remote work among a cohort of Finnish knowledge workers from 2019 to 2023. A specific aim was to examine whether the associations would differ before, during, or after the COVID-19 pandemic. We hypothesized that the rates of short SA would follow the changes in the amount of remote work, especially during and after the COVID-19 pandemic.

## Material and methods

For this register-based prospective study, we included all employees whose employers were signed up for our study and permitted to utilize employer-owned register data of SA and remote work (*N* = 7885, four Finnish organizations) for 2019–2023. In Finland, such register data do not require consent from employees, but following the European Union’s General Data Protection Regulation (2016/679), they were informed about the obtained data, the purposes of the data use, and they were provided the opportunity to decline the use of the data. Two of the organizations were from the public sector (approximately 450 and 1300 employees/year, respectively), and two were private sector enterprises (annual number of employees was 450–1050). All had a system to track working hours and locations daily. Employees worked in expert positions requiring a university-level degree or equivalent education. The final study sample was defined by limiting data to those with no missing data and at least 6 months of data in 2 consecutive years (*N* = 5535 employees).

The employers’ electronic records of daily working hours, work locations, and SAs from the beginning of 2019 to the end of 2023 were used as individual-level data. This data consisted of starting and ending times of daily working hours, working at the employer’s premises or remotely, travel for work, and the reasons for any absences (day off, sick leave, maternity leave, annual leave, etc.). SAs were identified from these records, but the SA did not include any health-related information, being limited to the start and end dates of SA spells. The working hours and locations were initially entered into the tracking system by the employees, but since the data are used as a basis for salary in each participating organization, it can be assumed as valid while it is comprehensive (i.e. no missing entries). While processing the data, all duplicate entries of working hours and locations were removed, and any overlapping entries were evaluated, that is, if there were two entries for a date, it was checked whether they were partial duplicates or subsequent entries (<5% of all observations). For partial duplicates, that is, with the same start or end time, or a difference in work location (premises vs. remote), we selected the first entry. For all subsequent entries, for example, a new start time for work after an already-entered end time, the limit of 1 h was used. Then, all subsequent entries with 1 h or less between them were collapsed into one entry, and the working hours for the earliest start and latest end were calculated. Remote work was systematically assigned for a date on which full or partial working hours were entered as remote work. Out of all remote workdays, 17% were partial remote workdays. In this study, we focused on the short (1–3 days of self-certified) SA and calculated the number of short SA days per week for all weeks in 2019–2023.

As a factor of interest, we calculated the number of days worked remotely per week based on the employer-owned register data. Thus, remote workdays/week were calculated for each week in 2019–2023. Then we utilized this measure both as continuous and categorical, classified into three categories: none; 1–2 days of remote work/week; or 3–5 days of remote work/week. The categorization was utilized to assess whether there might be a specific number of days that might provide a basis for practical solutions at workplaces to organize their work. Furthermore, daily and weekly working hours were estimated, daily working hours separately for remote workdays and in general for each week in 2019–2023.

For the sample, we also identified background characteristics that were available from the register data: age and sex for the year 2022.

This study was approved by the Ethical Board of the Finnish Institute of Occupational Health, Helsinki, Finland.

## Statistical analyses

Descriptive characteristics, including means with standard deviations (SD) or percentages (%) were calculated. The means 2019–2023 for remote workdays/week, working hours/day, remote working hours/days, and short SA days/week were estimated to visualize trends over time. Then, a conditional Poisson regression model with a longitudinal design and fixed-effects option (fe option) to account for the repeated nature of observations in the data was applied to obtain incidence rate ratios (IRRs) with 95% confidence intervals for associations between remote work and short SA. The benefit of using the fe option is that it is used only within individual observations that change over time. Thus, the fe option controls all observed time-invariant and unobserved time-invariant individual characteristics in the measurement period. We controlled the weekly working hours in the models as a measure of work participation and potential work overload, since they have been shown to play a role in remote work and SA [26-28]. Supplemental Table S1 includes results without controlling for the weekly working hours. We also utilized a time lag as a sensitivity analysis, where remote work (and working hours) was measured the week before SA. The results retained the associations of the main analyses (data not shown). The analyses were performed using Stata 18 MP.

## Results

**Figure 1.**
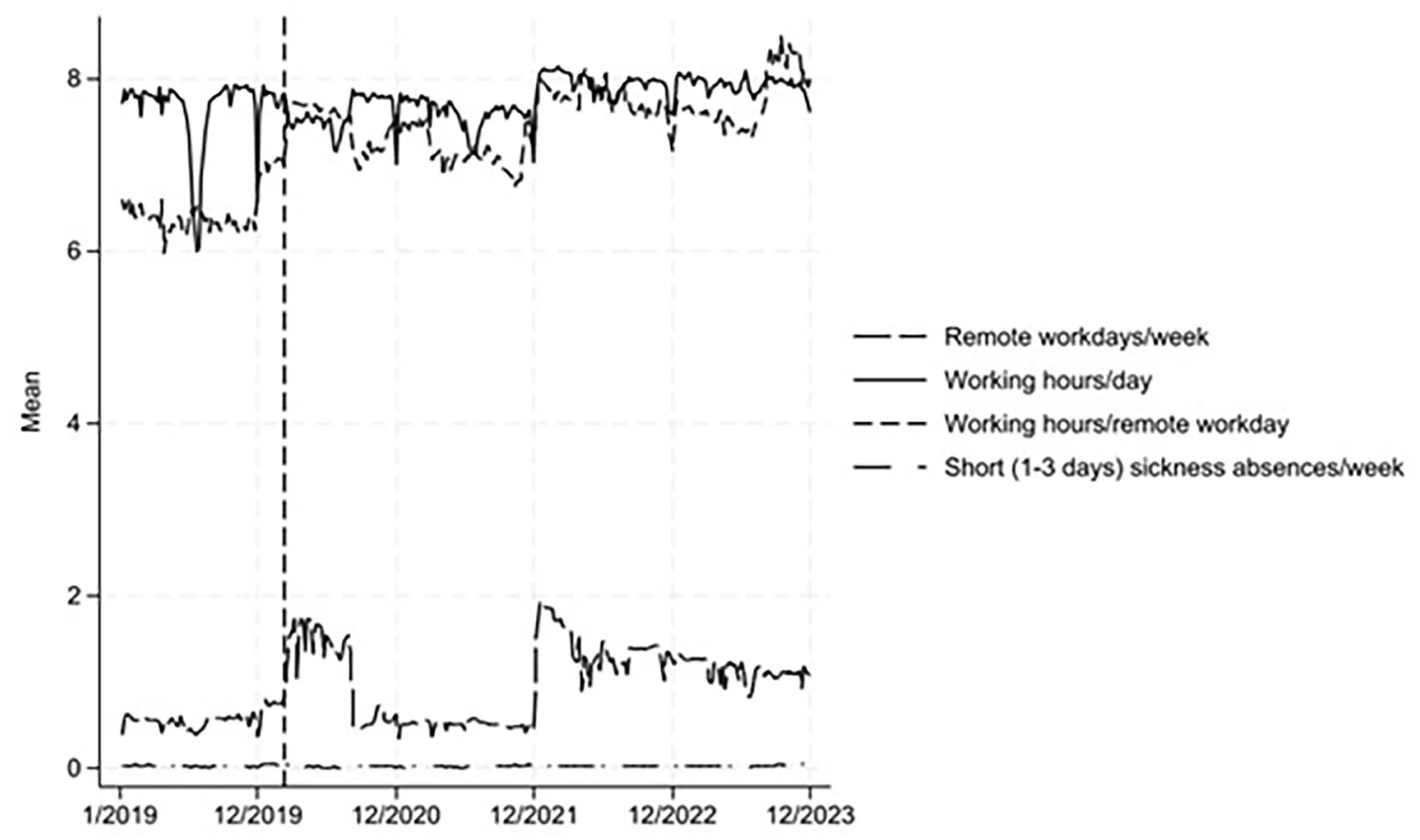
Weekly means of remote workdays and short (1–3 days) SA days, and average working hours/day (overall) and working hours/remote workday. SA, sickness absence.

First, we report the overall means for remote workdays, working hours, and short, 1–3 days, SAs across 2019–2023 for the final sample (Figure 1). Among this sample, only 4% had part-time work contracts, and 23% were men. The mean age in 2022 was 49 years (SD 9.3). In Figure 1, the lockdown in Finland in week 12 has been marked with a dashed vertical line. Regarding the short SA, the levels remained low throughout the study period. However, weekly remote workdays increased after the lockdown and again at the end of 2021 when most of the restrictions in Finland were removed. Daily working hours also seem rather stable during the study period, except that the working hours in remote working days have increased and stabilized at that level after the lockdown.

Second, we tested the associations between remote workdays/week with short SA (Table I) across the study period. Overall, each 1-day increase in remote workdays/week was associated with a higher likelihood of short SA, but categorizing the remote workdays into none, 1–2 days/week, or 3–5 days/week indicated that having no remote workdays was associated with lower odds of short SA, and 3–5 days with higher odds. Weekly working hours (Table I) showed a statistically significant association with SA.

**Table I.** The fixed-effects Poisson regression analysis for the association between the amount of remote work and short (1–3 days) sickness absences.

	*N* = 5535		
	%	IRR^#^	95%CI
Remote workdays/week (continuous)		**1.27**	**1.24, 1.30**
Categorized remote workdays/week			
None	43	**0.36**	**0.33, 0.40**
1–2 days/week	19	Ref	–
3–5 days/week	38	**1.46**	**1.34, 1.59**
Weekly working hours		**0.94**	**0.94, 0.94**

# The continuous and categorized remote workdays were analyzed in separate models. Both models were adjusted for the organization, and the result of working hours was the same in both models.

IRR, incidence rate ratios; 95%CI, 95% confidence interval; Ref, reference.

Statistically significant IRR with 95%CI in boldface.

To elaborate on these findings, we ran the models for each year of follow up separately (Table II), while accounting for the weekly working hours. There, the association for each 1-day increase in remote workdays attenuated to statistical insignificance except for the years 2020, where the association was in the opposite direction, and 2021. On the other hand, a closer look at each year and the categorized remote workdays/week indicated that the finding of no remote workdays being associated with lower odds of short SA was systematic across all years, except 2019, where the association was statistically non-significant. Instead, when we compared frequent remote work to none, both 1–2 days and 3–5 days/week were associated with a higher likelihood in 2021, 2022, and 2023. To emphasize the utilization of remote days’ categorization, we also tested the 3–5 remote workdays/week as a reference. In these results, no remote workdays were associated with a lower likelihood of short SA in 2021–2023. In 2019, none of the remote workday measures were related to short SA. Supplemental Table S1 indicates that when weekly working hours were not controlled for, the associations between the remote workdays/week as a continuous measure were consistently toward a lesser likelihood of short SA. The categorized measures of remote workdays/week followed the same pattern while controlling the weekly working hours (Supplemental Table S1 vs. Table II), having a greater magnitude.

**Table II.** The fixed-effects Poisson regression analysis for the association between the amount of remote work and short (1–3 days) sickness absences across the years 2019–2023 (data limited to those who had at least 2 consecutive years of data).

	2019		2020		2021		2022		2023	
	IRR^#^	95%CI	IRR^#^	95%CI	IRR^#^	95%CI	IRR^#^	95%CI	IRR^#^	95%CI
Remote workdays/week	0.99	0.82, 1.19	**0.93**	**0.87, 1.00**	**1.38**	**1.20, 1.59**	0.99	0.94, 1.04	0.95	0.90, 1.01
Categorized amount of remote workdays/week										
None	1.13	0.63, 2.03	**0.72**	**0.56, 0.93**	**0.57**	**0.35, 0.91**	**0.33**	**0.27, 0.41**	**0.33**	**0.26, 0.41**
1–2 days/week	Ref	–	Ref	–	Ref	–	Ref	–	Ref	–
3–5 days/week	1.02	0.46, 2.30	**0.62**	**0.45, 0.87**	**1.97**	**1.04, 3.75**	0.96	0.82, 1.12	0.95	0.80, 1.12
Categorized amount of remote workdays/week										
None	Ref	–	Ref	–	Ref	–	Ref	–	Ref	–
1–2 days/week	0.88	0.49, 1.59	**1.39**	**1.07, 1.80**	**1.77**	**1.10, 2.83**	**3.01**	**2.43, 3.73**	**3.03**	**2.44, 3.78**
3–5 days/week	0.90	0.41, 1.98	0.87	0.64, 1.18	**3.49**	**1.95, 6.22**	**2.89**	**2.25, 3.71**	**2.87**	**2.20, 3.75**
Categorized amount of remote workdays/week										
None	1.11	0.50, 2.43	1.15	0.85, 1.57	**0.29**	**0.16, 0.51**	**0.35**	**0.27, 0.44**	**0.35**	**0.27, 0.45**
1–2 days/week	0.98	0.44, 2.19	**1.60**	**1.15, 2.23**	**0.51**	**0.27, 0.96**	1.04	0.89, 1.22	1.06	0.90, 1.25
3–5 days/week	Ref	–	Ref	–	Ref	–	Ref	–	Ref	–

# Models adjusted for the weekly working hours.

IRR, incidence rate ratios; 95%CI, 95% confidence interval; Ref, reference.

Statistically significant IRR with 95%CI in boldface.

## Discussion

This prospective study of 5535 Finnish knowledge workers utilized employer-owned register data of remote work and short (1–3 days) SAs across 2019–2023. We aimed to elaborate on potential differences between years, as the COVID-19 pandemic was expected to affect both remote workdays and short SA. At the mean levels of short SA and remote workdays across these years, the rates and/or changes in rates were not simultaneous. Instead, for the associations between remote work and short SA, our results indicated that each 1-day increase in remote workdays/week (i.e. continuous measure) was associated with short SA in 2021–2022. However, the yearly analysis indicated that the categorized levels of remote workdays/week were associated with short SA in the years 2020–2023. Specifically, those without remote workdays (i.e. none per week) were less likely to have short SA, whereas those working 1–2 or 3–5 days/week remotely had a higher likelihood. Thus, our hypothesis that the rates of short SA would follow the changes in the amount of remote work, especially during and after the COVID-19 pandemic, was partially supported.

This study is among the first to utilize employer-owned daily and detailed register data of remote work, working hours, and SA to investigate knowledge workers longitudinally [15]. Thus, our results add to the existing knowledge that has been based on the preceding or early years of the pandemic only [11-13,29], or on cross-sectional design and/or survey data [3,8,12,14]. However, our results do not provide any specific limits for remote workdays/week. Still, instead, they highlight the fact that monitoring both remote work and short SA at workplaces could be a means to identify employees at risk for presenteeism, compromised well-being, or work overload. The specific feature of this study was to control the working hours in the associations between remote work and short SA. Supplemental Table S1 shows that the association, without accounting for the weekly working hours, follows the patterns but in a greater magnitude. Thus, such results might be indicative of working hours playing a role in the associations as assumed, based on earlier research, while they also may reflect the so-called healthy worker effect (i.e. those in good health may work longer, or more constant, working hours) [30]. This was also new, although earlier research on irregular working hours in healthcare and reviews of remote work have pointed toward the fact that the work participation or workload based on the working hours might be important [19,28].

The utilization of employer-owned register data of working hours and SA is a promising new way of research, as such data are free from reporting or memory biases and without loss to follow up [31-33]. The application of such data to remote work among knowledge workers has been scarce. However, due to increased rates of employees working remotely, there is an emergent need for studies that can follow and estimate both remote work, other work characteristics (such as working hours or employer premises), and health-related outcomes (such as SA, well-being, or job satisfaction) [14,15,19,34] simultaneously, to increase understanding of their complex associations that may vary across time.

Among the findings of this study, the fact that remote work was not systematically associated with short SA raises some speculations. First, since we observed a very stable, low amount of short SA from 2019 to 2023, but fluctuations in remote workdays at the same time, the implication might be that there are other influential factors related to an individual (such as age and sex) or organization (e.g. environment or instructions) for this association [7,10,35,36]. In this study, we cannot rule out the effects of organizational factors that may be related to remote work instructions, organizational culture, or location (i.e. accessibility using public transportation). However, the use of the fe model controlled for age, sex, and, for example, stable health status, and, at least to a small extent, organizational effects, as the individuals were compared with themselves. The second speculation builds on the assumption that knowledge workers may stay at home to work remotely even while sick [7,9]. We cannot rule out presenteeism, and since we observed differences between the amount of remote work in its association with short SA, and that doing no remote work was linked with less-likely short SA, this might hold. This aligns with survey studies about remote work and presenteeism [10,19]. Furthermore, since the findings seem to imply the most consistent associations between remote work and short SA in 2021–2023, this might be because of the lessons learned from the COVID-19 pandemic, that is, ‘stay home if you have symptoms of an infectious disease’. At least in Finland, such recommendations have been visible in occupational healthcare, workplaces, and even the media. If this assumption is correct, further studies to elaborate on this would be merited, but it would also suggest that remote work could be a solution to preventing contamination of infectious diseases at the workplace and even to add flexibility and work participation in knowledge work.

This study benefited from a longitudinal, prospective design using detailed register data of working hours, remote work, and SAs. The follow up, from 2019 to 2023, enabled investigation before the COVID-19 pandemic, and even after all restrictions were removed in 2022. Yet, we applied for fe Poisson regression to account for the repetitive nature of our panel data. This methodological solution also added rigor via the feature of controlling for all stable characteristics of the individuals (i.e. age, sex, organization), whether measured or not. Thus, our results were based on the within-individual variation, which might be an important issue because SAs are known to vary due to age, sex, and educational differences, but also organizational factors such as instructions and regulations may play a role, as they do for remote work [7,10]. Since we relied on register data only, we lacked further information on working conditions that could be assessed via surveys, such as job satisfaction, perceived privacy at work, or remote work circumstances [15,35,36]. Thus, further studies should elaborate on these issues. On the other hand, we only had data on those employees who stayed at these organizations for at least 6 months in 2 consecutive years during 2019–2023. Since our original sample (*N* = 7885) diminished to a final sample of 5535, there was a considerable loss to the follow up, which should add caution to the interpretation of our findings. The generalizability of these findings might be limited to those Nordic welfare states that share similar systems and possibilities for remote work, working hours, and SAs.

## Conclusions

Among Finnish knowledge workers, remote work seems to be associated with short, 1–3 days of SA only after the COVID-19 pandemic. The possibility of working remotely might be an important factor in mitigating infections, while assumptions exist that presenteeism might be prevalent in knowledge work that enables remote work.

## Supplemental Material

sj-docx-1-sjp-10.1177_14034948251380639 – Supplemental material for Trends and associations of remote workdays and short sickness absences among Finnish knowledge workers from 2019 to 2023 Supplemental material, sj-docx-1-sjp-10.1177_14034948251380639 for Trends and associations of remote workdays and short sickness absences among Finnish knowledge workers from 2019 to 2023 by Annina Ropponen and Annu Haapakangas in Scandinavian Journal of Public Health
