# Supplementary material for: Trends and associations of remote workdays and short sickness absences among Finnish knowledge workers from 2019 to 2023
Source: Scand J Public Health. 2025 Oct 7;54(7):753–9. doi: 10.1177/14034948251380639 (PMC13589794; doi:10.1177/14034948251380639)
Supplement: Supplementary file 1 [file sj-docx-1-sjp-10.1177_14034948251380639.docx]

|  | **2019** | | **2020** | | **2021** | | **2022** | | **2023** | |
| --- | --- | --- | --- | --- | --- | --- | --- | --- | --- | --- |
|  | **IRR^#^** | **95%CI** | **IRR^#^** | **95%CI** | **IRR^#^** | **95%CI** | **IRR^#^** | **95%CI** | **IRR^#^** | **95%CI** |
| Remote workdays/week | 0.97 | 0.81, 1.16 | **0.88** | **0.83, 0.94** | 0.99 | 0.87, 1.12 | **0.66** | **0.63, 0.69** | **0.62** | **0.59, 0.65** |
| Categorized amount of remote workdays/week |  |  |  |  |  |  |  |  |  |  |
| None | 0.69 | 0.39, 1.24 | **0.58** | **0.47, 0.73** | **0.49** | **0.32, 0.74** | **0.12** | **0.10, 0.15** | **0.18** | **0.14, 0.22** |
| 1-2 days/week | ref | - | ref | - | ref | - | ref | - | ref | - |
| 3-5 days/week | **9.66** | **5.07, 18.39** | **2.08** | **1.65, 2.62** | **4.30** | **2.76, 6.69** | **0.61** | **0.54, 0.70** | **0.64** | **0.57, 0.74** |
|  |  |  |  |  |  |  |  |  |  |  |
| Categorized amount of remote workdays/week |  |  |  |  |  |  |  |  |  |  |
| None | ref | - | ref | - | ref | - | ref | - | ref | - |
| 1-2 days/week | 1.44 | 0.80, 2.59 | **1.72** | **1.37, 2.15** | **2.05** | **1.34, 3.14** | **8.40** | **6.84, 10.31** | **5.66** | **4.64, 6.91** |
| 3-5 days/week | **13.95** | **10.00, 19.45** | **3.57** | **2.95, 4.32** | **8.84** | **7.08, 11.03** | **5.13** | **4.21, 6.26** | **3.65** | **3.01, 4.42** |
|  |  |  |  |  |  |  |  |  |  |  |
| Categorized amount of remote workdays/week |  |  |  |  |  |  |  |  |  |  |
| None | **0.07** | **0.05, 0.10** | **0.28** | **0.23, 0.34** | **0.11** | **0.09, 0.14** | **0.19** | **0.16, 0.24** | **0.27** | **0.23, 0.33** |
| 1-2 days/week | **0.10** | **0.05, 0.20** | **0.48** | **0.38, 0.61** | **0.23** | **0.15, 0.36** | **1.64** | **1.43, 1.87** | **1.55** | **1.36, 1.77** |
| 3-5 days/week | ref | - | ref | - | ref | - | ref | - | ref | - |

**Supplemental Table S1** The fixed effects Poisson regression analysis for the association between the amount of remote work and short (1-3 days) sickness absences across the years 2019-2023 (data limited to those who had at least 2 consecutive years of data)
